# Charge Self‐Regulation of Metallic Heterostructure Ni_2_P@Co_9_S_8_ for Alkaline Water Electrolysis with Ultralow Overpotential at Large Current Density

**DOI:** 10.1002/advs.202303682

**Published:** 2023-10-22

**Authors:** Xingxing Zhu, Xue Yao, Xingyou Lang, Jie Liu, Chandra‐Veer Singh, Erhong Song, Yongfu Zhu, Qing Jiang

**Affiliations:** ^1^ Key Laboratory of Automobile Materials Ministry of Education School of Materials Science and Engineering Jilin University 130022 Changchun China; ^2^ Department of Materials Science and Engineering University of Toronto Toronto ON M5S 3E4 Canada; ^3^ State Key Lab of High Performance Ceramics and Superfine Microstructure Shanghai Institute of Ceramics Chinese Academy of Sciences Shanghai 200050 China; ^4^ Department of Mechanical and Industrial Engineering University of Toronto Toronto ON M5S 3G8 Canada; ^5^ Center of Materials Science and Optoelectronics Engineering University of Chinese Academy of Sciences Beijing 100049 China

**Keywords:** active electronic state, bifunctional electrocatalyst, heterostructure, one‐step self‐assembly approach, water splitting

## Abstract

Designing cost‐effective alkaline water‐splitting electrocatalysts is essential for large‐scale hydrogen production. However, nonprecious catalysts face challenges in achieving high activity and durability at a large current density. An effective strategy for designing high‐performance electrocatalysts is regulating the active electronic states near the Fermi‐level, which can improve the intrinsic activity and increase the number of active sites. As a proof‐of‐concept, it proposes a one‐step self‐assembly approach to fabricate a novel metallic heterostructure based on nickel phosphide and cobalt sulfide (Ni_2_P@Co_9_S_8_) composite. The charge transfer between active Ni sites of Ni_2_P and Co─Co bonds of Co_9_S_8_ efficiently enhances the active electronic states of Ni sites, and consequently, Ni_2_P@Co_9_S_8_ exhibits remarkably low overpotentials of 188 and 253 mV to reach the current density of 100 mA cm^−2^ for the hydrogen evolution reaction and oxygen evolution reaction, respectively. This leads to the Ni_2_P@Co_9_S_8_ incorporated water electrolyzer possessing an ultralow cell voltage of 1.66 V@100 mA cm^−2^ with ≈100% retention over 100 h, surpassing the commercial Pt/C║RuO_2_ catalyst (1.9 V@100 mA cm^−2^). This work provides a promising methodology to boost the activity of overall water splitting with ultralow overpotentials at large current density by shedding light on the charge self‐regulation of metallic heterostructure.

## Graphical abstract

The metallic Ni_2_P@Co_9_S_8_ heterostructure is a highly efficient and stable bifunctional catalyst for overall water splitting. The electron transfer from Co─Co bonds of Co_9_S_8_ to active Ni sites of Ni_2_P in the heterointerface modulates the active electronic states of Ni sites, holding great potential to lower the energy cost for hydrogen generation.

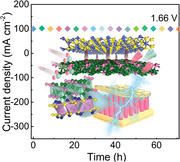

## 1 Introduction

Electrochemical water splitting is a sustainable and promising technology for clean hydrogen generation, especially in alkaline conditions that are commercially practical for large‐scale hydrogen production.^[^ ^1^ ^]^ While commercial Pt/C and IrO_2_/RuO_2_ have demonstrated exceptional activity for hydrogen evolution reaction (HER) and oxygen evolution reaction (OER), respectively, their high cost and limited availability hinder their widespread applications.^[^ ^2^ ^]^ Recently, nonprecious metal‐based catalysts exhibit remarkable electrocatalytic activity for water splitting at low current density (10‐50 mA cm^−2^), but achieving larger current density for large‐scale production remains a challenge.^[^ ^3^ ^]^ To address this issue, various approaches such as bimetallic engineering,^[^ ^4^, ^5^ ^]^ crystal phase regulation,^[^ ^6^ ^]^ defect introduction,^[^ ^7^, ^8^ ^]^ nonmetal doping,^[^ ^9^ ^]^ and heterostructure construction^[^ ^10^, ^11^ ^]^ have been extensively developed. Based on the earlier pioneering work,^[^ ^12^, ^13^, ^14^, ^15^ ^]^ the sluggish water dissociation step in alkaline media has been identified as a bottleneck for robust water electrolysis with low cell voltage. This involves the Volmer step of cleaving H─OH bond to form an adsorbed hydrogen atom (*H*_ad_) and the Heyrovsky step of combining *H*_ad_ with other H─OH bonds to generate H_2_. Thus, water adsorption/dissociation and hydroxyl groups (OH^−^) adsorption lead to higher cell voltage at large current density.

To develop ideal bifunctional catalysts with large current density water splitting (HER/OER) at lower overpotentials, several requirements need to be fulfilled, including sufficient active sites for rapid adsorption/dissociation of OH^−^/H_2_O, high electrical conductivity, continuous H_2_/O_2_ escape, and superior mechanical stability. Ni‐based compounds, such as (oxy)hydroxides, oxides, sulfides, pnictides, nitrides, and their composites, have shown great potential for large current density water splitting.^[^ ^7^, ^11^, ^16^ ^]^ While Raney Ni has demonstrated potential as a bifunctional electrocatalyst for large current density water splitting, its intrinsic activity remains unsatisfactory, resulting in a cell voltage (1.8–2.4 V) that is significantly higher than the thermodynamic potential (1.23 V).^[^ ^14^ ^]^ It is highly desirable to design efficient Ni‐based catalysts for hydrogen production.^[^ ^17^ ^]^ Among various Ni‐based materials, nickel phosphide is a promising catalyst for HER due to its low cost and efficient catalytic activity.^[^ ^18^ ^]^ However, it has high energy barrier for oxygen‐containing intermediates adsorption in OER process and H_2_O molecule dissociation during the HER process in alkaline solution, which hinders its wide application in overall water splitting.^[^ ^19^ ^]^ Combining the nickel phosphide with other materials that need small energy to adsorb oxygen‐containing intermediates, such as oxides, hydroxides, and chalcogenides, is an effective strategy to derive advanced catalysts. Some novel heterostructures have been developed, such as Ni_5_P_4_@NiCo_2_O_4_,^[^ ^20^ ^]^ Co_2_P–Ni_2_P/TiO_2_^[^ ^21^ ^]^ and sc‐Ni_2_P^δ−^/NiHO,^[^ ^22^ ^]^ in which the oxides or hydroxides were used to expedite the kinetics of water splitting. Especially, the low conductivity of oxides and hydroxides hamper the electron transport during the electrochemical processes, leading to sluggish kinetics in the overall water splitting. Chalcogenides with higher electron conductivity are more suitable for use in nickel phosphide‐based heterostructure, although the S─H_ad_ bonds on the surface of the metal sulfides in the HER process are too strong, making the conversion of H_ad_ to H_2_ difficult. Thus, constructing heterostructure through combining chalcogenide equipped with higher electron conductivity with moderate H atom binding energy of nickel phosphide may be a promising approach to accelerate electron transfer kinetics and enhance overall water splitting ability.^[^ ^23^ ^]^

Herein, we design a novel bifunctional electrocatalyst via a one‐step self‐assembly strategy, consisting of Ni_2_P@Co_9_S_8_ metallic heterostructure conductor attached to a vulcanized nickel foam (VNF). The unique structure of Ni_2_P@Co_9_S_8_ metallic heterostructure provides increased exposure of active sites to electrolytes and enables efficient adsorption/desorption of *H and oxygen‐contain intermediates while lowering the energy barrier for water dissociation. As a result, the as‐prepared catalyst exhibits remarkably low overpotentials of 188 and 253 mV to reach the current density of 100 mA cm^−2^ of HER and OER, respectively. In a symmetrical two‐electrode cell, Ni_2_P@Co_9_S_8_ performs effectively as both the cathode and anode, achieving an ultralow cell voltage of only 1.66 V at the current density of 100 mA cm^−2^ for overall water splitting. This approach provides a promising method for the design of bifunctional electrocatalysts for water splitting.

## 2 Results and Discussion

**Figure 1:**
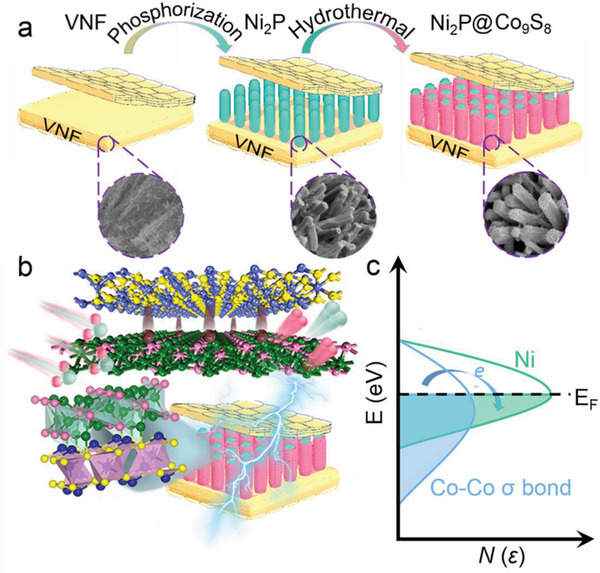
Schematic diagrams. Schematic illustrations of a) the fabrication of Co_9_S_8_ nanoflakes on Ni_2_P nanorods, b) the water splitting of Ni_2_P@Co_9_S_8_ metallic heterostructure, and c) the charge compensation effect on the active electronic states in Ni_2_P@Co_9_S_8_ metallic heterostructure.

As depicted schematically in **Figure** **1**a, the synthesis of a Co_9_S_8_ nanoflakes‐anchored Ni_2_P nanorods structure was achieved through one‐step self‐assembly hydrothermal method (see Experimental Section for details). The resulting metallic heterointerface between Ni_2_P and Co_9_S_8_, which can be used for overall water splitting, is further depicted in Figure 1b. In Figure 1c, we propose that the Co_9_S_8_ containing active Co─Co σ bonds could serve as an electron reservoir to regulate the electronic state of active Ni_2_P. For the Ni_2_P@Co_9_S_8_ composite, the charge regulation from Co─Co σ bonds to an active site can further optimize the active electronic states of Ni around the Fermi‐level, favorable for enhancing catalytic activity. Gap states around the Fermi‐level are mainly generated by active Ni atoms, where the charge of active atoms is transferred between Co─Co bonds and Ni atoms. The activated 3d‐bands of Ni atom in Ni_2_P@Co_9_S_8_ can not only interact strongly with S atom in deep energy level but also isolate across the Fermi‐level benefitting the formation of heterointerface and the enhanced catalytic activity of OER half‐reaction. Thus, the charge regulation effect on the active electronic states of Ni and Co sites around the Fermi‐level, in which the Co─Co regulate the Ni active sites near the Fermi‐level, which form moderate electronic states for intermediates adsorption in the electrochemical reaction, resulting in improved electrocatalytic performance. In addition, morphology analysis was carried out using field‐emission scanning electron microscopy (FESEM), transmission electron microscopy (TEM), and high‐angle annular dark‐field scanning transmission electron microscopy (HAADF‐STEM).

**Figure 2:**
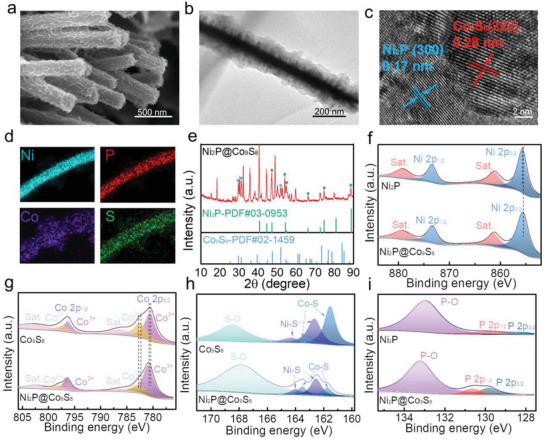
Characterizations of VNF supported catalysts. a) FESEM image, b) TEM image, and c) HRTEM image of Ni_2_P@Co_9_S_8_. d) Elemental mapping images of Ni, P, Co, and S and e) XRD pattern of Ni_2_P@Co_9_S_8_. XPS of f) Ni 2p for Ni_2_P@Co_9_S_8_ and Ni_2_P, g) Co 2p for Ni_2_P@Co_9_S_8_ and Co_9_S_8_, h) S 2p for Co_9_S_8_ and Ni_2_P@Co_9_S_8_ and i) P 2p for Ni_2_P@Co_9_S_8_ and Ni_2_P.

As shown in **Figure** **2**a, the SEM image of Ni_2_P@Co_9_S_8_ demonstrates the uniform anchoring of Co_9_S_8_ nanoflakes on Ni_2_P nanorods, consistent with the individual Ni_2_P nanorods and Co_9_S_8_ nanoflakes shown in Figure S1 (Supporting Information). Notably, the Ni_2_P nanorods, generated from VNF as a nickel source, seamlessly contact the foam, which promotes overall electron transmission of the integrated material and benefits charge transfer at the Ni_2_P@Co_9_S_8_ interface. The TEM image in Figure 2b further confirms the coexistence of Ni_2_P nanorods and Co_9_S_8_ nanoflakes, with the nanoflakes partially distributed at the edges of nanorods, consistent with the diameter size of FESEM results. The high‐resolution TEM (HRTEM) image in Figure 2c shows clear lattice fringes, with spacing lattices of 0.28 and 0.17 nm, corresponding to the (222) plane of Co_9_S_8_ crystal and (300) plane of Ni_2_P crystal respectively, consistent with the results of Figure S2 (Supporting Information), verifying the successful synthesis of the Ni_2_P@Co_9_S_8_ metallic heterostructure. The elemental mapping of Ni_2_P@Co_9_S_8_ is determined by HAADF‐STEM (Figure 2d), revealing the homogeneous distribution of Ni, P, Co, and S elements within the metallic heterostructure. As depicted in Figure 2e, apart from the VNF, the peaks emerging at 2θ = 29.7°, 31.2°, and 51.9° are indexed to the (311), (222) and (440) planes of Co_9_S_8_ (JCPDS file No. 02–1459), respectively. The peaks located at 44.5°, 47.3°, 54.2°, 54.9°, 66.2°, 72.6°, 74.6°, and 88.8° can be ascribed to (201), (210), (300), (211), (310), (311), (400), and (321) planes of Ni_2_P (JCPDS file No. 03–0953), respectively. The X‐ray diffraction (XRD) patterns of contrast samples are displayed in Figure S3 (Supporting Information).

To further investigate the chemical states and elemental composition of the composites, X‐ray photoelectron spectroscopy (XPS) analysis was conducted (Figure 2f–i). For Ni_2_P@Co_9_S_8_, the Ni 2p_3/2_ and Ni 2p_1/2_ peaks located at 855.4 and 873.6 eV with two satellites are fitted, indicating the Ni^2+^ oxidation state. Compared to Ni_2_P,^[^ ^24^ ^]^ the Ni 2p peak is negatively shifted by 0.2 eV. Additionally, as depicted in Figure 2g, the Co 2p signals located at about 780.6 eV in Co 2p_3/2_ and 796.7 eV in Co 2p_1/2_ can be indexed to the spin‐orbit characteristics of Co^3+^, while the peaks at about 782.6 eV in Co 2p _3/2_ and 797.5 eV in Co 2p_1/2_ are attributed to the characteristics of Co^2+^. Compared to the sample of Co_9_S_8_,^[^ ^25^ ^]^ the XPS peaks of Co 2p of Ni_2_P@Co_9_S_8_ are respectively positively shifted by 0.3 eV, indicating a strong interaction between Co_9_S_8_ and Ni_2_P. Ni─S and Co─S bonds can be observed in Figure 2h. The down‐shift of Ni 2p and up‐shift of Co 2p binding energy reveal the electron transfer from Co_9_S_8_ to Ni_2_P, demonstrating the charge redistribution and strong electronic interactions among Ni_2_P nanorods and Co_9_S_8_ nanoflakes, which is beneficial to decreasing transfer resistance (*R*_ct_) and promoting the catalytic properties. The characterized peaks of P 2p in Ni_2_P@Co_9_S_8_ also positively shift toward higher binding energy compared to Ni_2_P (Figure 2i).^[^ ^24^ ^]^ The shift of Ni and Co binding energy leads to the increase of the valence density, beneficial to the reaction with oxygen intermediates.

**Figure 3:**
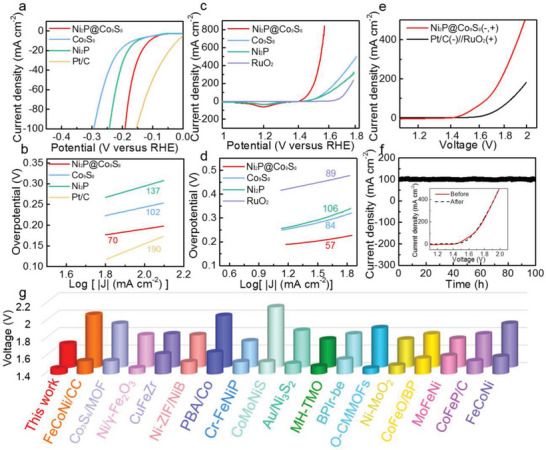
Catalytic performances of VNF supported catalysts. a) LSV curves with *iR*‐compensation and b) Tafel slopes of Ni_2_P@Co_9_S_8_, Co_9_S_8_, Ni_2_P and Pt/C for HER in 1 m KOH. c) LSV curves with *iR*‐compensation and d) Tafel slopes of Ni_2_P@Co_9_S_8_, Co_9_S_8_, Ni_2_P and RuO_2_ for OER in 1 m KOH. e) LSV curves for electrocatalytic overall water splitting in 1 m KOH. f) The *i‐* *‐t* curve of Ni_2_P@Co_9_S_8_//Ni_2_P@Co_9_S_8_ at cell voltage of 1.66 V for 100 h. Inset: LSV curves before and after the durability measurement. g) Comparison of working voltage of Ni_2_P@Co_9_S_8_//Ni_2_P@Co_9_S_8_ and related alkaline electrolyzer at the current density of 10 and 100 mA cm^−2^ with those reported previously.

The electrocatalytic HER behavior of all as‐prepared samples was investigated in 1 M KOH solution using three‐electrode system. In **Figure** **3**a and Figure S4 (Supporting Information), the linear sweep voltammetry (LSV) curves of Ni_2_P@Co_9_S_8_ with those of pure NF, VNF, Ni_2_P, Co_9_S_8_ and Pt/C‐NF at scan rate of 5 mV s^−1^ were compared. Ni_2_P@Co_9_S_8_ just delivers the smallest overpotential of 188 mV to reach the current density of 100 mA cm^−2^, indicating the significant effect of the cooperation Co_9_S_8_ nanoflakes and Ni_2_P nanorods on HER performance. The superior HER performance of Ni_2_P@Co_9_S_8_ can also be manifested through the Tafel slopes based on obtained LSV curves. As shown in Figure 3b and Figure S5 (Supporting Information), the Tafel slope value of Ni_2_P@Co_9_S_8_ is as low as 70 mV dec^−1^, much lower than those of contrast samples, suggesting more favorable reaction kinetics of Ni_2_P@Co_9_S_8_ towards HER. The mechanism of Ni_2_P@Co_9_S_8_ electrode can be considered as Volmer‐Heyrovsky process.

The values of double‐layer capacitance (*C*_dl_) were used to further assess the electrochemical active surface area (ECSA) in the non‐Faradic potential interval. As shown in Figures S6 and S7 (Supporting Information), the Ni_2_P@Co_9_S_8_ possesses the highest *C*_dl_ value using cyclic voltammetry (CV) method of 91 mF cm^−2^, significantly larger than those of other catalysts, indicating that Ni_2_P@Co_9_S_8_ can achieve increased area density. To further confirm this, the roughness factor (RF) values were also assessed by dividing the determined *C*_dl_ by 40 µF cm^–2^, which was usually regarded as *C*_dl_ value of an ideally flat electrode in an alkaline solution,^[^ ^26^ ^]^ and the results are shown in Table S1 (Supporting Information). The fact that the Ni_2_P@Co_9_S_8_ has the largest ECSA and RF values among these contrast samples suggests the high exposure of this hybrid structure, which is beneficial to sufficient contact with the electrolyte and enhanced H_2_O molecule adsorption ability. In addition, to eliminate the contribution from the roughness factor, as exhibited in Figure S8 (Supporting Information), the LSV curves were normalized with their corresponding ECSA values, consistent with Figure 3a, indicating the superior intrinsic activity of Ni_2_P@Co_9_S_8_.

Electrochemical impedance spectroscopy (EIS) was utilized to gain insights into the reaction kinetics. As shown in Figure S9 (Supporting Information), the as‐prepared catalysts formed semicircles in the plane plots. Based on the equivalent circuit (inset of Figure S9, Supporting Information), the *R*_ct_ value of Ni_2_P@Co_9_S_8_ is 2.79 Ω, which is lower than those of contrast samples (Table S2, Supporting Information). This observation indicates faster charge‐transfer kinetics and high efficiency of reaction at the interface of Ni_2_P@Co_9_S_8_ during the HER process. This improved performance may be attributed to the integration of Ni_2_P nanorods seamless on VNF, electron transfer from Co_9_S_8_ to Ni_2_P, and the more homogeneous distribution of Co_9_S_8_ nanoflakes on Ni_2_P nanorods. Furthermore, to evaluate the electrochemical HER durability of the Ni_2_P@Co_9_S_8_ structure, an *i‐* *t* curve was recorded at the fixed potential of −0.188 V versus RHE for over 100 h in 1 m KOH (Figure S10, Supporting Information). There is no palpable attenuation in its corresponding current density of −100 mA cm^−2^, highlighting the superior structural stability during the HER process. In addition, the remarkable stability of Ni_2_P@Co_9_S_8_ as an electrode for HER was further confirmed by the LSV curves before and after 100 h test (inset of Figure S10, Supporting Information).

In addition to its excellent HER performance, the activity of Ni_2_P@Co_9_S_8_ towards OER was evaluated in the same electrolyte. As shown in Figure 3c and Figure S11 (Supporting Information), the acquired overpotential to obtain 100 mA cm^−2^ of Ni_2_P@Co_9_S_8_ is 253 mV, lower than the cases of Co_9_S_8_ (335 mV), Ni_2_P (333 mV), and VNF (387 mV) and other reported work,^[^ ^4^, ^9^, ^10^, ^11^, ^12^, ^18^ ^]^ indicating superior OER activity. In Figure 3d and Figure S12 (Supporting Information), the small Tafel slope value of 57 mV dec^−1^ for Ni_2_P@Co_9_S_8_ is lower than those values of Ni_2_P, Co_9_S_8_, VNF, NF, and RuO_2_‐NF, reflecting its fast reaction kinetics and superior OER catalytic performance. Based on the CV curves (Figure S13, Supporting Information), as shown in Figure S14 (Supporting Information), the Ni_2_P@Co_9_S_8_ shows the largest *C*_dl_ value of 50 mF cm^−2^ among these samples, indicating that it can provide the largest electrochemical active surface areas and the most accessible active sites for the OER reaction. The RF values based on *C*_dl_ values are depicted in Table S3 (Supporting Information), and the LSV curves after normalization are exhibited in Figure S15 (Supporting Information), suggesting the outstanding intrinsic activity of Ni_2_P@Co_9_S_8_ for the OER process. In addition, Ni_2_P@Co_9_S_8_ exhibits the smallest *R*_ct_ as 2.51 Ω in Figure S16 and Table S4 (Supporting Information) among the as‐prepared samples, implying remarkable reaction kinetic in the OER process. In this work, the formation of metallic heterogeneous Ni_2_P@Co_9_S_8_ further reduced the activation energy of chemical reactions and accelerated the interfacial charge transfer dynamics, which is beneficial to optimizing intermediate adsorption, decreasing the *R*_ct_ value and promoting the catalytic performance. This is consistent with the results of Tables S2 and S4 (Supporting Information).

As depicted in Figure S17 (Supporting Information), the Ni_2_P@Co_9_S_8_ demonstrates exceptional electrochemical durability for 100 h at fixed potential of 1.485 V versus RHE. Notably, the current density after 100 h has only a negligible attenuation. In the inset of Figure S17 (Supporting Information), moreover, there exists ignorable variation in the LSV curves between the original one and the one after 100 h test at 100 mA cm^−2^, which further confirms the stability of the structure. Based on the above electrochemical properties, the Ni_2_P@Co_9_S_8_ possesses a more exposed active site, enhanced intrinsic electrocatalytic performance, and favorable reaction kinetics, which strongly suggests that the Co_9_S_8_ can modulate the electron distribution of Ni_2_P, beneficial to achieving remarkable HER and OER behavior. The contrast samples, in contrast, require larger overpotentials than Ni_2_P@Co_9_S_8_ to attain high current densities for HER and OER, indicating that Ni_2_P@Co_9_S_8_ possesses excellent catalytic behavior.

To evaluate its catalytic activity for overall water decomposition, we assembled two‐electrode equipment using Ni_2_P@Co_9_S_8_ as both the anode and cathode, as it showed outstanding electrocatalytic properties for the HER and OER in an alkaline solution. As seen in Figure 3e, the Ni_2_P@Co_9_S_8_||Ni_2_P@Co_9_S_8_ can reach a current density of 10 mA cm^−2^ at 1.46 V, surpassing the commercial Pt/C║RuO_2_ catalyst. Additionally, the voltage window only slightly dropped after 100 h of electrolysis at 100 mA cm^−2^ (Figure 3f). After 100 h durability measurements, the LSV curve exhibits minimal change compared to the original one, indicating the stability of the structure (inset of Figure 3f). All these show that Ni_2_P@Co_9_S_8_ possesses attractive stability.

After the electrochemical process, as depicted in Figure S18a, (Supporting Information), the XRD pattern obtained after HER showed unobvious changes, suggesting the chemical stability during HER. As to the XRD pattern after OER, the characterization peaks are the same as the initial sample, but the intensity of some peaks located at 18.4°, 29.7°, 44.5°, and 51.9° is weaker than the initial sample, which may be attributed to the partial phase transition during OER. At the same time, to further illustrate the structural reconstruction during OER, we tested in‐situ Raman patterns at different voltages (Figure S18b, Supporting Information), and the characteristic peaks located at around 460 and 550 cm^−1^ effectively show that oxyhydroxides are formed during OER.^[^ ^27a^ ^]^ The charge regulation mechanism should be still applicable to reconstructed catalysts by modulating the active electronic states of active sites. In addition, the charge regulation of pre‐catalysts can optimize the electronic states of reactive species, which is beneficial for the structure reconstruction of catalysts during OER.^[^ ^6^, ^12^, ^27^ ^]^ As presented in Tables S5–S7 (Supporting Information) and Figure 3g, the Ni_2_P@Co_9_S_8_ electrode exhibits superior parameters of Tafel slope and overpotentials for HER and OER and thus of cell voltage for overall water splitting performance, outperforming most reported non‐precious metal‐based electrocatalytic materials.^[^ ^27^, ^28^, ^29^, ^30^, ^31^, ^32^, ^33^ ^]^ Commonly, in order to meet the requirements for electrolysis of water under industrial alkaline conditions, the current density should achieve 200–400 mA cm^−2^ at the cell voltage of 1.8–2.4 V.^[^ ^34^ ^]^ The Ni_2_P@Co_9_S_8_ just needs a cell voltage of 1.76 V to achieve the current density of 200 mA cm^−2^, outperforming Pt/C║RuO_2_ (1.99 V), suggesting Ni_2_P@Co_9_S_8_ can lower the energy cost during the hydrogen production than commercial catalyst. Besides, to reach the current density of 200 mA cm^−2^_,_ Ni_2_P@Co_9_S_8_ only needs 204 and 276 mV for HER and OER, respectively. We have compared the overpotentials required to achieve the current density of 200 mA cm^−2^ for HER, OER, and overall water splitting with reported catalysts (Tables S8–S10, Supporting Information). The results show that Ni_2_P@Co_9_S_8_ holds great potential for industrial water electrolysis.

**Figure 4:**
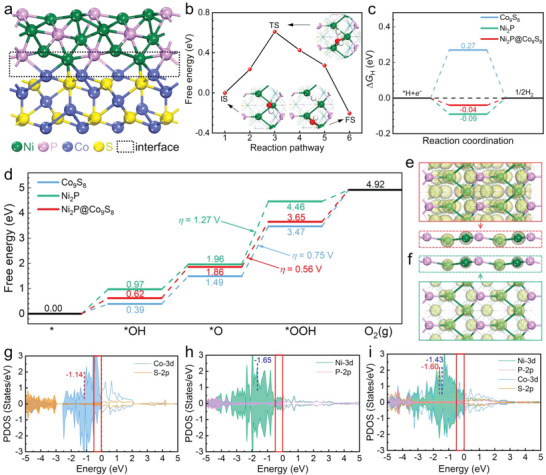
DFT analyses. a) Optimized structure of Ni_2_P@Co_9_S_8_. b) The energy barrier and reaction pathway of water dissociation on Ni_2_P@Co_9_S_8_. Insets are configurations of initial state (IS), transition state (TS), and final state (FS). Colar code: O, red; H, white. Free energy profiles of (c) HER and (d) OER on Co_9_S_8_, Ni_2_P, and Ni_2_P@Co_9_S_8_. Charge density analysis of e) Ni_2_P@Co_9_S_8_ and f) Ni_2_P at an isovalue of 0.15 *e* Å^−1[.3]^ Dashed boxes side views of top layers for visual comparison between surface‐active Ni sites in Ni_2_P@Co_9_S_8_ and Ni_2_P. Projected density of state (PDOS) of g) Co_9_S_8_, h) Ni_2_P, and i) Ni_2_P@Co_9_S_8_, including the corresponding *ε*_d_ values.

To investigate the outstanding HER and OER activities of Ni_2_P@Co_9_S_8_, the dissociation of water and adsorption of reaction intermediates on Ni_2_P, Co_9_S_8_, and Ni_2_P@Co_9_S_8_ were thoroughly examined using density functional theory (DFT) calculations (see **Figure** **4**a; Figure S19, Supporting Information for models). In alkaline media, the kinetic rate of HER process is primarily governed by the energy barrier induced by the water dissociation process. As shown in Figure 4b, the energy barrier of water dissociation on the active Ni sites of Ni_2_P@Co_9_S_8_ (0.61 eV) is lower than that of Ni_2_P (0.79 eV, Figure S20, Supporting Information) and Co_9_S_8_ (0.99 eV, Figure S21, Supporting Information), suggesting that the heterostructure accelerates water dissociation for faster protons generation. Besides the water decomposition, the subsequent hydrogen adsorption also plays a vital role in determining the apparent alkaline HER activity, and the optimal ∆*G*_H_ value is 0 eV. Figure 4c shows the free energy profile of HER and Figure S22 (Supporting Information) provides the corresponding configurations. Compared to Ni_2_P (0.27 eV) and Co_9_S_8_ (−0.09 eV), the ∆*G*_H_ value of Ni_2_P@Co_9_S_8_ (−0.04 eV) is much closer to 0 eV, suggesting that the heterostructure exhibits enhanced HER activity.

Regarding the OER activity, the ∆*G* values of reaction intermediates (*OH, *O, and *OOH) are effective descriptors. According to the free energy profile shown in Figure 4d and the configurations of *OH, *O, and *OOH shown in Figure S22 (Supporting Information), the elementary step of *O oxidation (*O + OH^−^ → *OOH + *e* ^−^) is the rate‐determining step (RDS) for both Ni_2_P and Co_9_S_8_, with ∆*G*_RDS_ = 2.50 and 1.98 eV, respectively. Compared to Ni_2_P, interestingly, the active Ni sites remain the optimal active sites for Ni_2_P@Co_9_S_8_ but with much strong adsorption of reaction intermediates. The RDS is the elementary step of *O oxidation as well, but the corresponding ∆*G*_RDS_ decreases to 1.79 eV. Consequently, the overpotential of OER of Ni_2_P@Co_9_S_8_ (*η* = 0.56 V) is lower than that of Co_9_S_8_ (*η* = 0.75 V) and Ni_2_P (*η* = 1.27 V), indicating that the heterostructure exhibits enhanced OER activity.

The unique electronic structure of Ni_2_P@Co_9_S_8_ is responsible for its optimal ∆*G*_H_ value and also for its ∆*G*_RDS_ and *η* values lower than pure Ni_2_P and Co_9_S_8_. Since both Ni_2_P and Ni_2_P@Co_9_S_8_ have the same active Ni sites, charge density analysis was conducted on the surface active Ni sites to see the inherent difference (Figure 4e,f). In reference to Ni_2_P, the charge densities of Ni sites in Ni_2_P@Co_9_S_8_ are clearly larger, suggesting the electronic regulation of Ni_2_P due to the formation of heterostructure. Such a difference can be further confirmed by the Bader charge analysis (Figure S23, Supporting Information), where smaller Bader charge values and thus lower oxidation state of active Ni sites in Ni_2_P@Co_9_S_8_ can be observed. This agrees well with the XPS results shown in Figure 2f that the valence state of Ni atoms in Ni_2_P@Co_9_S_8_ is lower than that in Ni_2_P. Accordingly, the reduction states of P and S atoms should also change to maintain the electric neutrality of the whole system. To further clarify the charge transfer, the Bader charge analysis of the topmost Co and S atoms in Co_9_S_8_ and Ni_2_P@Co_9_S_8_ was performed (Figure S24, Supporting Information). With Ni_2_P@Co_9_S_8_ formation, the average Bader charge of three topmost Co atoms changes from +0.37|e| to +0.47|e|, while it changes from +0.48|e| to +0.52|e| for three topmost S atoms. This indicates that the charge variation of non‐metal atoms should be relatively negligible in comparison with mental atoms. For a deeper understanding, the PDOS figures of Ni_2_P, Co_9_S_8_, and Ni_2_P@Co_9_S_8_ are depicted. From Figure 4g,i, a significant charge density redistribution can be observed in Ni_2_P@Co_9_S_8_, qualitatively illustrating the redistribution of electronic states induced by the charge transfer. In Figure 4i, the strong hybridization between Co‐3d and P‐2p orbital can be found in the deep energy level (≈−3.5 eV), contributing to the formation of a metallic heterostructure of Ni_2_P@Co_9_S_8_. Moreover, around the Fermi level (−0.5–0 eV, red boxes), the active electronic states of active Ni sites in Ni_2_P@Co_9_S_8_ are highly increased along with the increased *d*‐band center (*ε*_d_; from −1.65 to −1.43 eV) compared to Ni_2_P (Figure 4h), suggesting more active electrons in Ni_2_P of Ni_2_P@Co_9_S_8_, which is highly conductive to optimizing the charge transfer and then improving the water splitting performance. Meanwhile, owing to the electronic regulation between Co─Co bonds and active Ni atoms, the active electronic states of Co sites around the Fermi level decrease, and *ε*_d_ decreases from −1.14 to −1.60 eV. Hence, compared to Ni_2_P and Co_9_S_8_, the Co─Co bonds in Ni_2_P@Co_9_S_8_ can regulate the intrinsic charge distribution of active Ni sites to further optimize the HER/OER performance.

## 3 Conclusion

In summary, we design a bifunctional electrocatalyst of Ni_2_P@Co_9_S_8_ composite conductor to perform highly efficient alkaline water electrolysis with ultralow overpotential at large current density. The constructed Ni_2_P@Co_9_S_8_║Ni_2_P@Co_9_S_8_ electrolyzer achieved an ultralow cell voltage of 1.66 V at a current density of 100 mA cm^−2^ and maintained operation stability over 100 h, which just requires low overpotentials of 188 and 253 mV to achieve HER and OER processes at the current density of 100 mA cm^−2^ in alkaline condition, respectively. The active electronic states of Ni around the Fermi‐level are modulated in the Ni_2_P@Co_9_S_8_ metallic heterostructure by the Co─Co bond, which contributes to the enhanced overall water‐splitting performance. Remarkably, the ultralow cell voltage of 1.66 V to achieve 100 mA cm^−2^ is contributed from the enhanced accessible surface area, faster electron transitions, and complementary function of different components of the Ni_2_P@Co_9_S_8_ metallic heterostructure. Our work provides a convenient and concise strategy for the design of large current‐density transition metal‐based catalysts in overall water splitting.

## 4 Experimental Section

### Synthesis of VNF, Ni_2_P, Co_9_S_8_, and Ni_2_P@Co_9_S_8_ Electrocatalysts

***]*** Typically, NF (30 mm × 20 mm × 1 mm) was treated through cleaning sequentially with solvents of acetone, 3 m HCl solution, distilled water, and absolute ethanol to ensure a clean surface.

After washing, the NF was immersed in the solution containing 0.076 g thiourea (1 mmol) and methanol (20 ml) in an oven at 180 °C for 6 h. The obtained sample was washed thoroughly with water and then placed in a vacuum oven at 60 °C for 8 h. After cooling down the autoclave at room temperature, the VNF was placed in 1 m KOH solution for 3 h, there will exist Ni(OH)_2_ nanoflakes upon VNF. After that, the Ni(OH)_2_ supported on VNF was placed in a tube furnace, with 0.5 g NaH_2_PO_2_ placed at the upstream side and near the above tube furnace. After flushing with argon gas, the furnace was heated up to 300 °C at a ramping rate of 1°C min^−1^. When the heating process was over, the temperature of 300 °C was held for 30 min to convert Ni(OH)_2_ to Ni_2_P. Then the Ni_2_P on VNF was submerged in a 100 mL Teflon‐lined stainless autoclave filled with 0.0474 g CoCl_2_·6H_2_O, 0.0496 g Na_2_S_2_O_3_ and 40 mL H_2_O and then heated in an oven at 150°C for 12 h. The resulting material was further treated with distilled water three times and dried in a vacuum at 60 °C. Finally, the Ni_2_P@Co_9_S_8_ sample was obtained. In addition, the VNF was directly immersed in 40 mL deionized water containing 0.0474 g CoCl_2_·6H_2_O and 0.0496 g Na_2_S_2_O_3_ in an oven at 150 °C for 12 h to obtain Co_9_S_8_. For the Pt/C and RuO_2_ electrodes, the catalysts (2 mg) were dispersed in a mixture of 960 µL ethanol and 40 µL Nafion solution (20 wt%), respectively. The 1 mL Pt/C and RuO_2_ inks were dropped into NF to obtain Pt/C‐NF and RuO_2_‐NF. The loading mass of Pt/C, RuO_2_, and Ni_2_P@Co_9_S_8_ are 1.91, 1.93, and 1.87 mg cm^−2^, respectively.

### Material Characterization

FESEM (JEOL JSM‐6700F, 15 keV) and TEM (JEOL JEM‐2100F, 200 keV) were used to further make sure the morphology and microstructure of as‐prepared electrodes. To analyze the material phase and crystallinity, XRD patterns were measured with the assistance of D8 FOCUS (powder) and D/max 2500 pc diffractometer (bulk) with Cu Kα radiation. XPS was collected using an ESCALAB Mk II (vacuum generator) spectrometer with an Al anode. All binding energies were calibrated via referencing to C 1*s* binding energy (284.8 eV). The Raman spectroscopy characterization was obtained with a Renishaw Raman spectrometer using an intensity of 0.05 mW with a wavelength of 532 nm as the exciting source.

### Electrochemical Characterization

The electrochemical characterization was carried out in a conventional three electrode electrochemical cell consisting of a graphite rod as a counter electrode, a standard calomel electrode (SCE) as the reference electrode and working electrode, and in alkaline solution (1 m KOH pH = 13.6) controlled by the CHI Instruments 660E electrochemical workstation. For all electrochemical measurements, the obtained SCE potential was converted into RHE based on the following formula: *E*_RHE_ = *E*_SCE_ + 0.244 + 0.0592 ×  pH. All of the data were *iR*‐calibrated in view of the equation: *E*_compensated_ = *E*_measured_‐ *iR*_s_ (*R*_s_ is the solution resistance based on the result of EIS). LSV curves of HER and OER processes were recorded at a scan rate of 5 mV s^−1^, and the scan rate of LSV curves for overall water splitting is 1 mV s^−1^. The ECSA was characterized by double‐layer capacitances based on CV curve measurements under different scan rates. The EIS spectra were recorded with fixed overpotentials set at −0.26 V versus RHE for HER and 1.53 V versus RHE for OER with the frequency range from 10^[^ ^5^ ^]^ to 10^−3^ H_Z_ (amplitude of 10 mV). The *i*–*t* curves were recorded for 100 h at a constant applied potential of −0.188 V versus RHE for HER and 1.485 V versus RHE for OER, respectively.

### Computational Details

Spin‐polarized DFT calculations were performed within the Vienna Ab initio Simulation Package (VASP), using the projector augmented wave (PAW) method, the generalized gradient approximation of Perdew‐Burke‐Ernzerhof (GGA‐PBE) functional, and the plane‐wave basis set with an energy cutoff of 450 eV.^[^ ^35^ ^]^ The Brillouin zones were sampled by a gamma‐centered 2 × 4 × 1 k point mesh, while a dense 6 × 12 × 1 mesh was used for electronic property calculations. The convergence criteria of structure optimization were set to be 10^−4^ eV for the electronic energy and 0.02 eV Å^−1^ for the forces on each atom. The slab models of Co_9_S_8_(111) and Ni_2_P(100) were built to simulate pure Co_9_S_8_ and Ni_2_P (Figure S19, Supporting Information), respectively. The mismatch between Co_9_S_8_(111) and Ni_2_P(100) is ≈3.6%, and subsequently, the Ni_2_P(100)@Co_9_S_8_(111) slab was constructed to explore the HER and OER activity of the Ni_2_P@Co_9_S_8_ heterostructure (Figure 4a). For all slab models, the vacuum space over 15 Å was added along the *z*‐axis to eliminate the interactions between neighboring models. The DFT‐D3 method was selected for the van der Waals correction.^[^ ^36^ ^]^ The minimum energy paths and transition states (TS) were determined by the climbing image nudged elastic band (CI‐NEB) method implemented in VASP.^[^ ^37^ ^]^

The Gibbs free energy of the intermediates (*H, *OH, *O, *OOH) during the HER and OER process was determined by,

(1) $$\Delta G=E_{\mathrm{ads}}+\Delta E_{\mathrm{ZPE}}-T\Delta S$$

where *E*_ads_ is the adsorption energy of the intermediate, ∆*E*_ZPE_ is the zero‐point energy difference between the adsorption and gas states, *T* is the temperature (298.15 K), and ∆*S* is the entropy change between the adsorption and gas phases. For intermediates, ∆*E*_ZPE_ and *S* were obtained from the vibrational frequency calculations with harmonic approximation, and the vibrational modes of intermediates were computed with the fixed slabs. For H_2_ and H_2_O molecules, ∆*E*_ZPE_ was computed in a 14×15×16 Å^[^ ^3^ ^]^ cell, while *S* at 298.15 K was taken from the handbook.^[^ ^38^ ^]^

The changes in the adsorption energy for *H, *OH, *O and *OOH were computed on the basis of DFT ground state energies as,

(2) $$\Delta E_{*H}=E\left(*H\right)-E\left(*\right)-1/2E\left(H_{2}\right)$$

(3) $$\Delta E_{*\mathrm{OH}}=E\left(*\mathrm{OH}\right)-E\left(*\right)+1/2E\left(H_{2}\right)-E\left(H_{2}O\right)$$

(4) $$\Delta E_{*O}=E\left(*O\right)-E\left(*\right)+E\left(H_{2}\right)-E\left(H_{2}O\right)$$

(5) $$\Delta E_{*\mathrm{OOH}}=E\left(*\mathrm{OOH}\right)-E\left(*\right)+3/2E\left(H_{2}\right)-2E\left(H_{2}O\right)$$

where * denotes the adsorption site on a substrate surface.

The OER process in an alkaline medium usually follows:

(6) $$*+{\mathrm{OH}}^{-}\to *\mathrm{OH}+e^{-},\;\Delta G_{1}$$

(7) $$*\mathrm{OH}+{\mathrm{OH}}^{-}\to *O+H_{2}O+e^{-},\;\Delta G_{2}$$

(8) $$*O+{\mathrm{OH}}^{-}\to *\mathrm{OOH}+e^{-},\;\Delta G_{3}$$

(9) $$*\mathrm{OOH}+{\mathrm{OH}}^{-}\to *+O_{2}+H_{2}O+e^{-}\;\Delta G_{4}$$

In addition,

(10) $$\Delta G_{1}=\Delta G_{*\mathrm{OH}}$$

(11) $$\Delta G_{2}=\Delta G_{*O}-\Delta G_{*\mathrm{OH}}$$

(12) $$\Delta G_{3}=\Delta G_{*\mathrm{OOH}}-\Delta G_{*O}$$

(13) $$\Delta G_{4}=4.92-\Delta G_{*\mathrm{OOH}}$$

The overpotential (*η*), an important parameter to evaluate the OER activity, was computed as,

(14) $$\eta =\max\{\Delta G_{1},\Delta G_{2},\Delta G_{3},\Delta G_{4}\}-1.23$$

## Conflict of Interest

The authors declare no conflict of interest.

## Author Contributions

X.Z. and X.Y. contributed equally to this work. Y.Z., E.S. conceived the research. X.Z. carried out the synthesis and performed materials characterization and electrochemical measurements. E.S. proposed the active electronic states and X.Y. carried out the theoretical calculations. Y.Z, E.S., C.S., and Q.J. wrote the manuscript. All authors discussed the results and commented on the manuscript.

## Supporting information

Supporting Information

## Data Availability

The data that support the findings of this study are available from the corresponding author upon reasonable request.
