# Supplementary material for: Charge Self‐Regulation of Metallic Heterostructure Ni2P@Co9S8 for Alkaline Water Electrolysis with Ultralow Overpotential at Large Current Density
Source: Adv Sci (Weinh). 2023 Oct 22;10(33):2303682. doi: 10.1002/advs.202303682 (PMC10667855; doi:10.1002/advs.202303682)
Supplement: Supplementary file 1 — Supporting Information [file ADVS-10-2303682-s001.pdf]

ADVANCED SCIENCE
Open Access

Supporting Information

for *Adv. Sci.*, DOI 10.1002/advs.202303682

Charge Self-Regulation of Metallic Heterostructure $Ni_2P@Co_9S_8$ for Alkaline Water Electrolysis with Ultralow Overpotential at Large Current Density

Xingxing Zhu, Xue Yao, Xingyou Lang, Jie Liu, Chandra-Veer Singh, Erhong Song*, Yongfu Zhu* and Qing Jiang**

Supporting Information

for

Charge Self-regulation of Metallic Heterostructure $Ni_2P@Co_9S_8$ for Alkaline Water Electrolysis with Ultralow Overpotential at Large Current Density

Xingxing Zhu[#], Xue Yao[#], Xingyou Lang, Jie Liu, Chandra-Veer Singh[*], Erhong Song[*], Yongfu Zhu[*], Qing Jiang[*]

X. Zhu, Prof. X. Lang, Prof. Y. Zhu, Prof. Q. Jiang

Key Laboratory of Automobile Materials, Ministry of Education, School of Materials Science and Engineering, Jilin University, Changchun 130022, China

E-mail: yfzhu@jlu.edu.cn

E-mail: jiangq@jlu.edu.cn

Prof. J. Liu, Prof. E. Song

State Key Lab of High Performance Ceramics and Superfine Microstructure, Shanghai Institute of Ceramics, Chinese Academy of Sciences, Shanghai, 200050, China

Prof. E. Song

Center of Materials Science and Optoelectronics Engineering, University of Chinese Academy of Sciences, Beijing, 100049, China

E-mail: ehsong@mail.sic.ac.cn;

Dr. X. Yao, Prof. C.-V. Singh

Department of Materials Science and Engineering, University of Toronto, Toronto, ON M5S 3E4, Canada

Email: chandraveer.singh@utoronto.ca

Prof. C.-V. Singh

Department of Mechanical and Industrial Engineering, University of Toronto, Toronto, ON M5S 3G8, Canada

#These authors contributed equally

*Corresponding authors

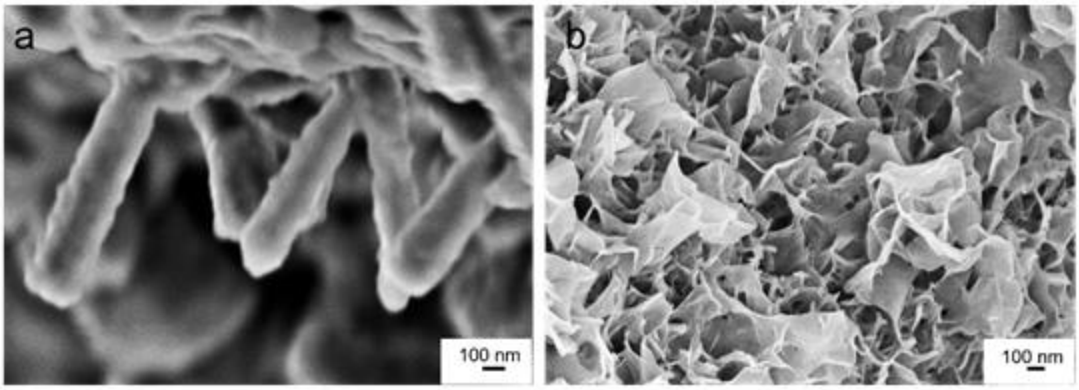

Figure S1 FESEM observation of (a) Ni_2P and (b) Co_9S_8.

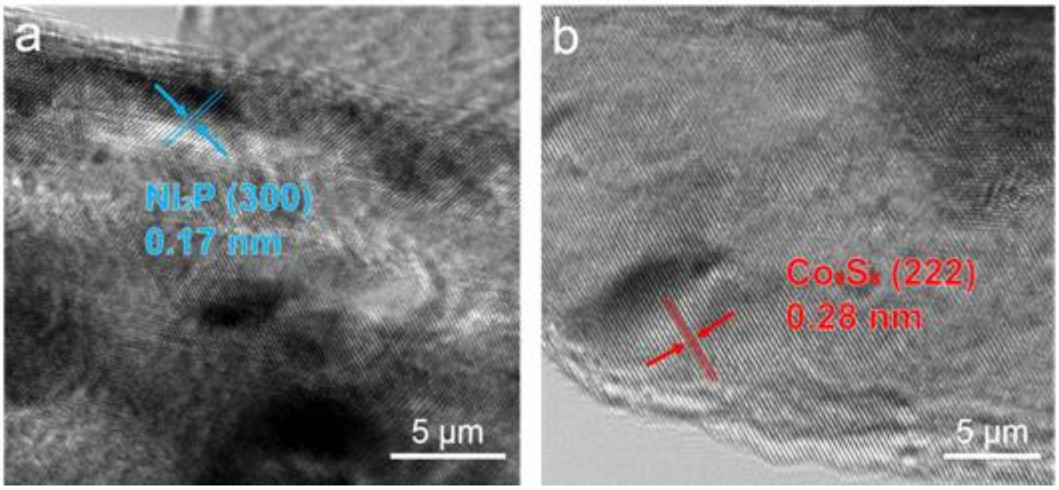

Figure S2 HR-TEM observation of (a) Ni_2P and (b) Co_9S_8.

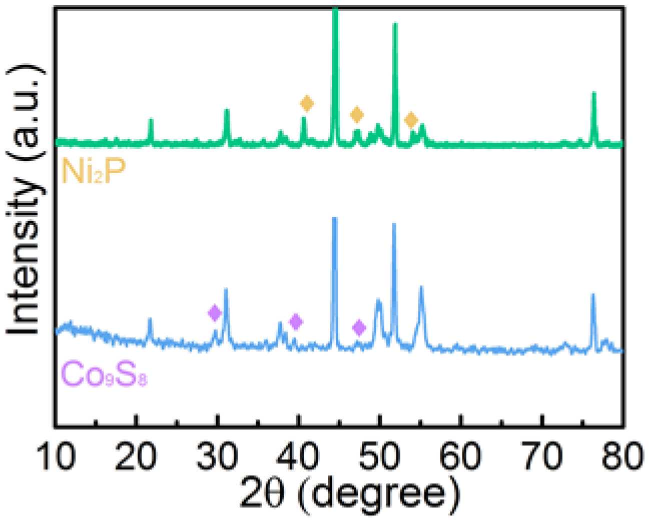

Figure S3 XRD patterns of Ni_2P and Co_9S_8.

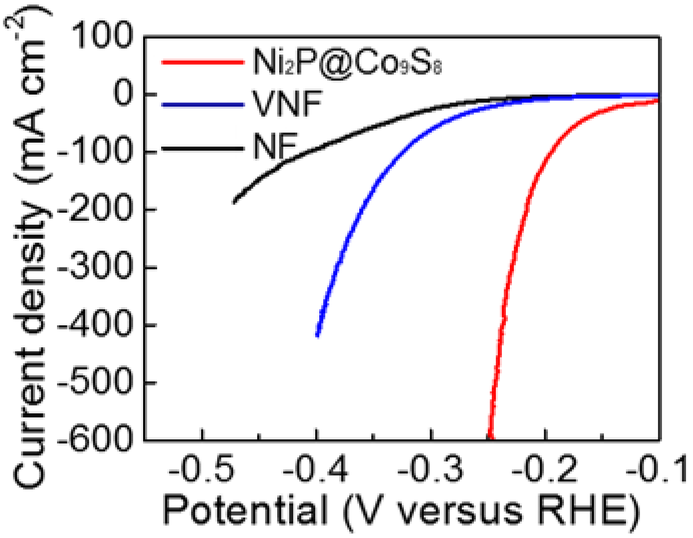

Figure S4 LSV curves of $Ni_2P@Co_9S_8$, VNF and NF for HER.

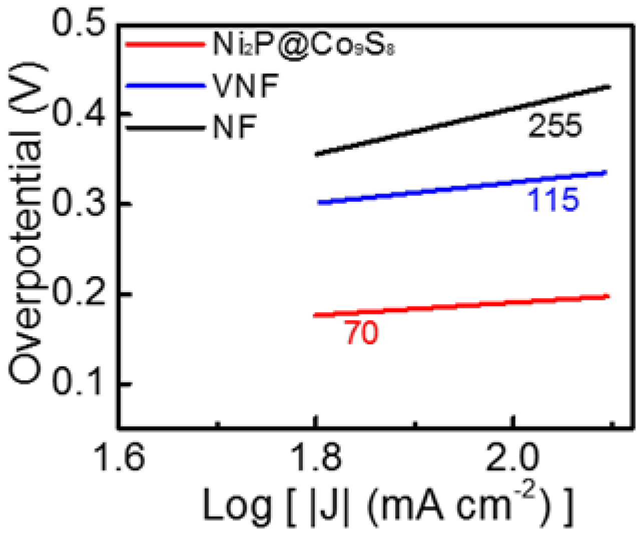

Figure S5 Tafel slopes of $Ni_2P@Co_9S_8$, VNF and NF for HER.

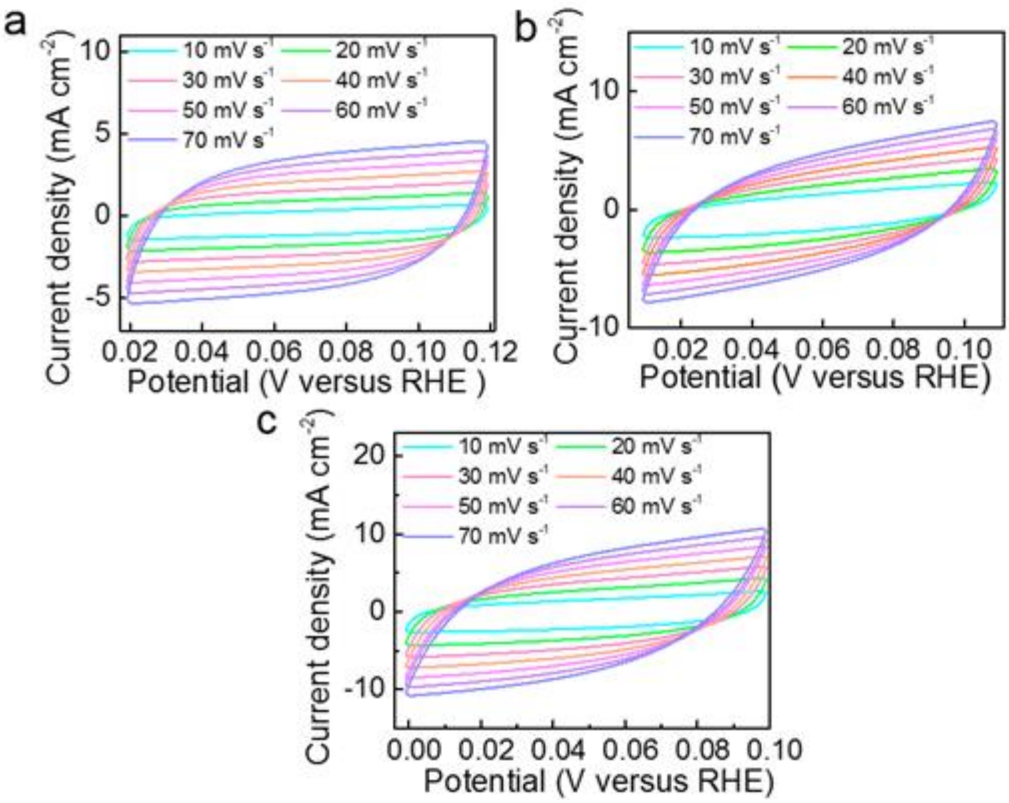

Figure S6 CV curves of (a) Ni_2P, (b) Co_9S_8 and (c) $Ni_2P@Co_9S_8$ for HER.

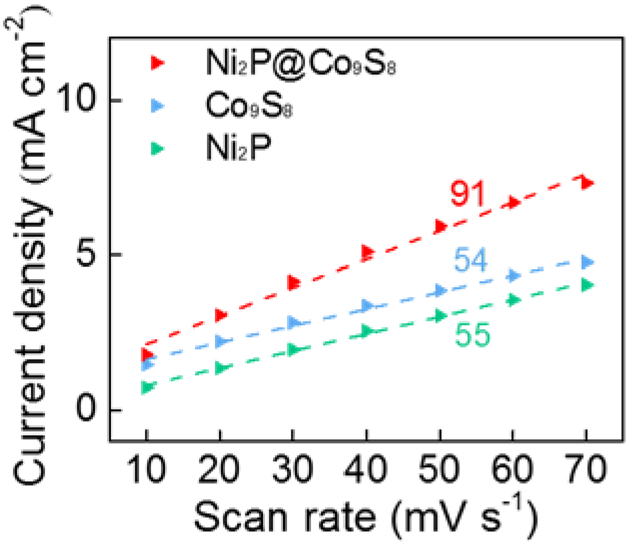

Figure S7 Plots used to evaluate the double-layer capacitances of Ni_2P, Co_9S_8 and $Ni_2P@Co_9S_8$ for HER.

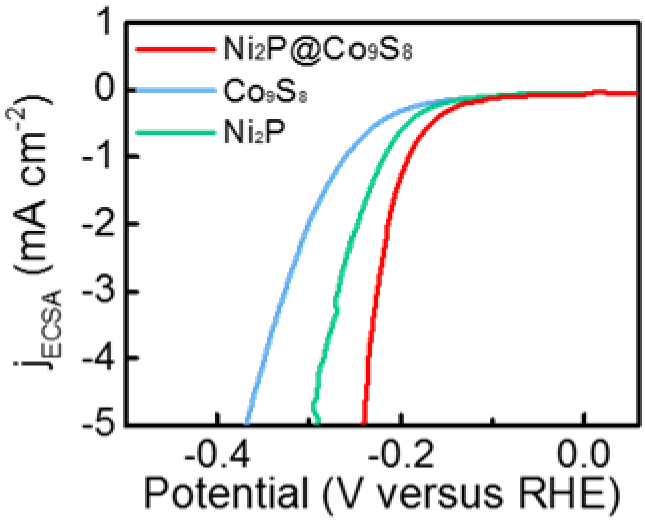

Figure S8 Polarization curves with the current density normalized to ECSA of Ni_2P, Co_9S_8 and $Ni_2P@Co_9S_8$ for HER.

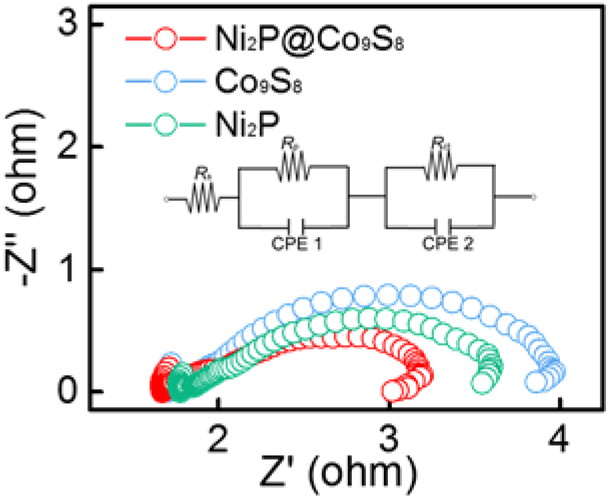

Figure S9 Nyquist plots of Ni_2P, Co_9S_8 and $Ni_2P@Co_9S_8$ for HER.

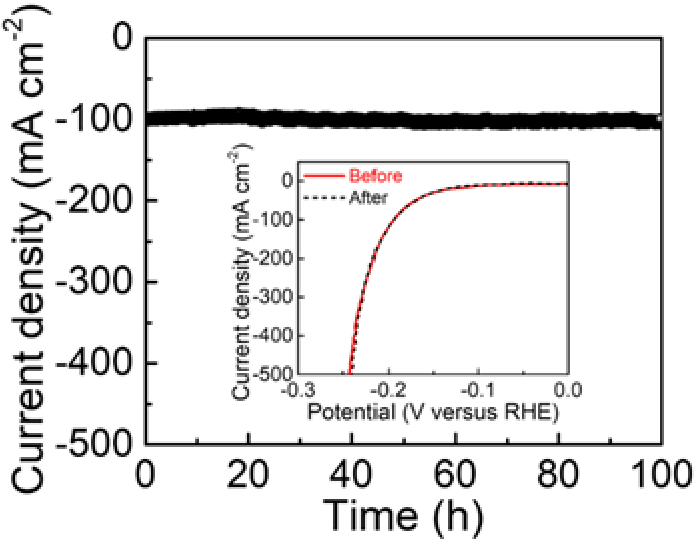

Figure S10 The long-time current density vs time curve (*i*-*t* curve) of $Ni_2P@Co_9S_8$ at the potential of -0.188 V versus RHE, and the inset shows the polarization data for $Ni_2P@Co_9S_8$ before and after 100 h.

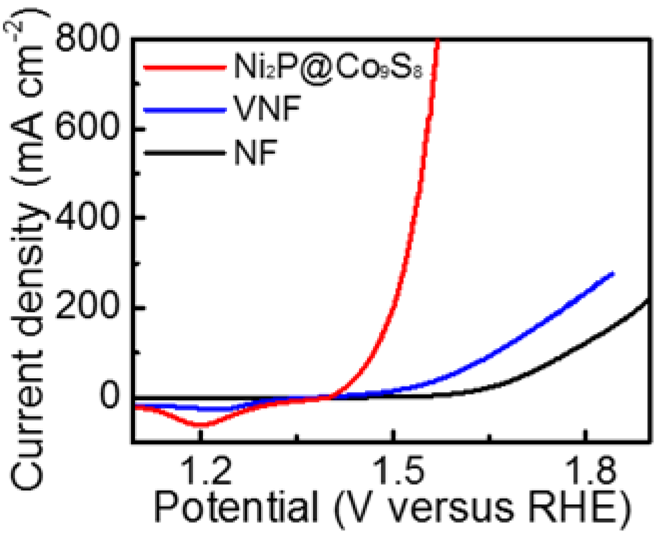

Figure S11 Polarization curves of $Ni_2P@Co_9S_8$, VNF and NF for OER.

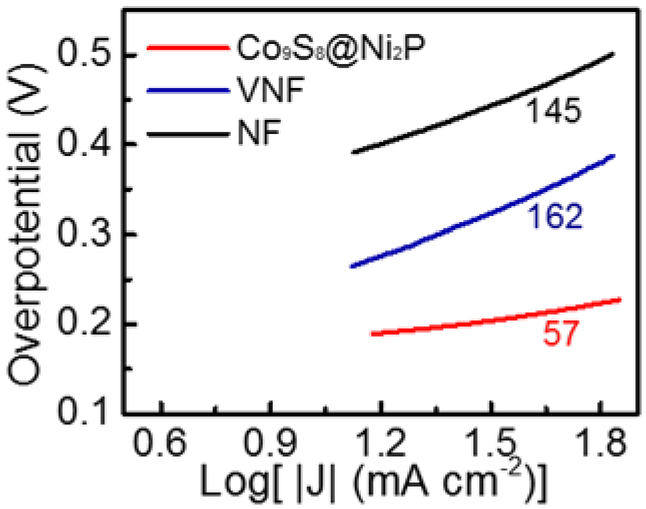

Figure S12 Tafel slopes of $Ni_2P@Co_9S_8$, VNF and NF for OER.

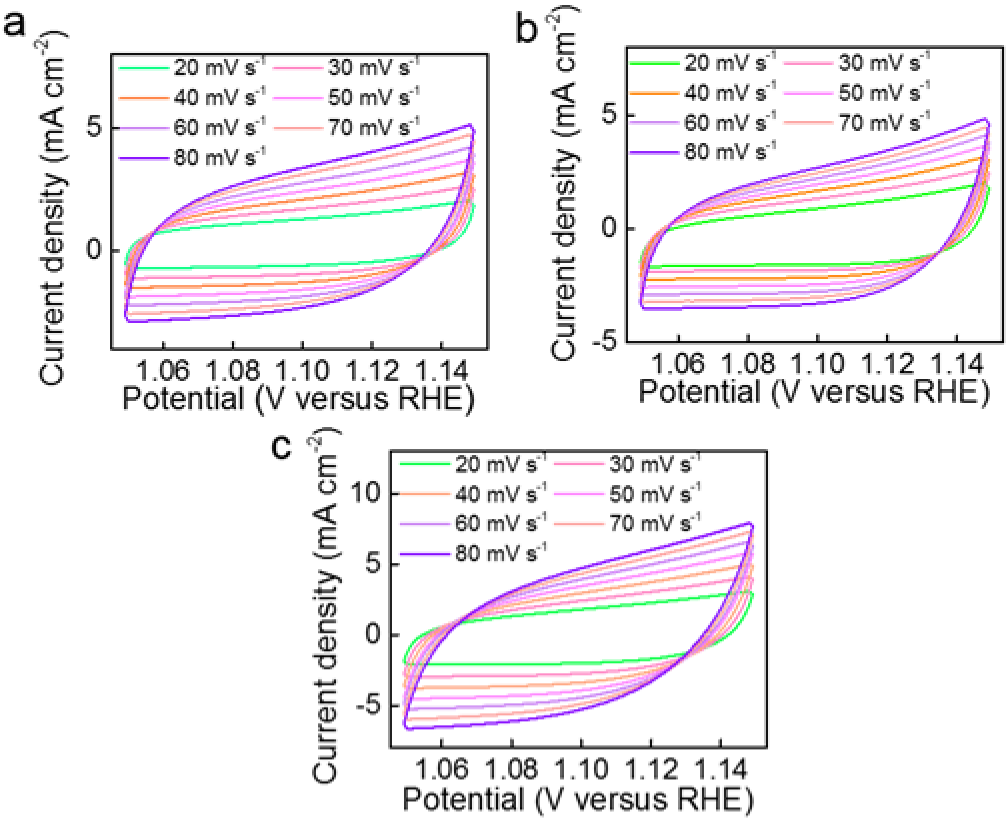

Figure S13 CV curves of (a) Ni_2P, (b) Co_9S_8 and (c) $Ni_2P@Co_9S_8$ for OER.

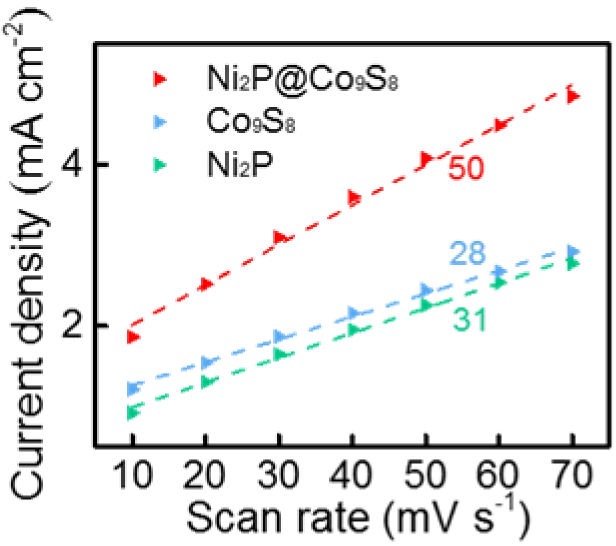

Figure S14 Plots used to evaluate the double-layer capacitances of Ni_2P, Co_9S_8 and $Ni_2P@Co_9S_8$ for OER.

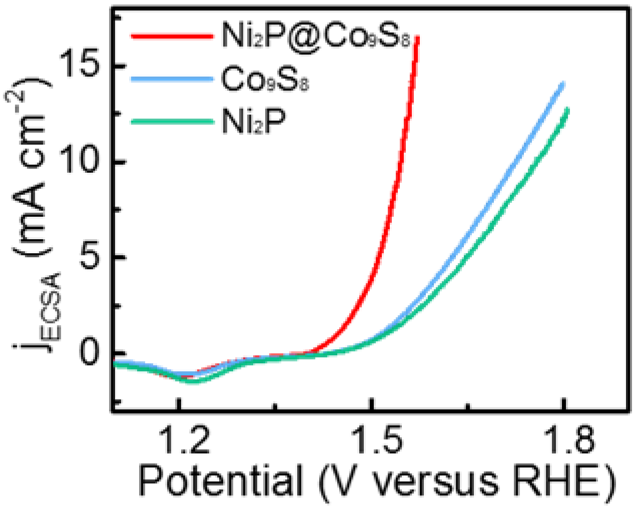

Figure S15 Polarization curves with the current density normalized to ECSA of Ni_2P, Co_9S_8 and $Ni_2P@Co_9S_8$ for OER.

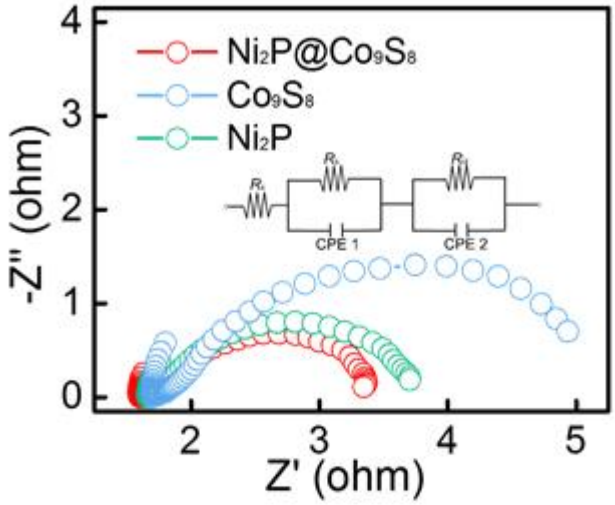

Figure S16 Nyquist plots of Ni_2P, Co_9S_8 and $Ni_2P@Co_9S_8$ for OER.

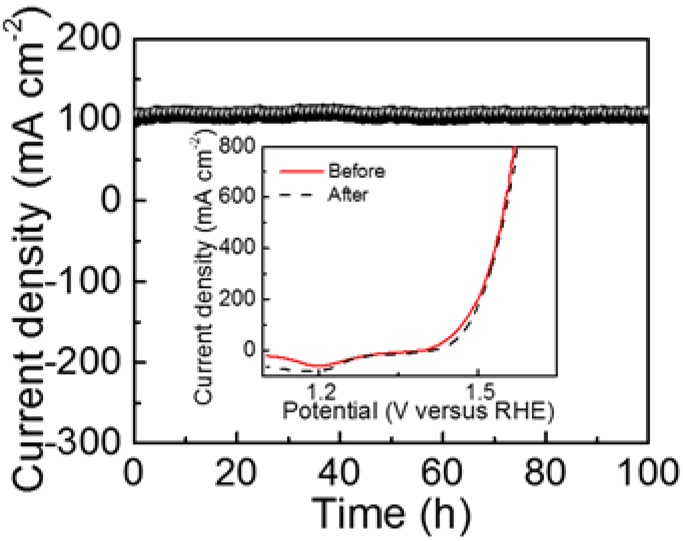

Figure S17 The *i-t* curve of $Ni_2P@Co_9S_8$ at the potential of 1.485 V versus RHE, and the inset shows the polarization data for $Ni_2P@Co_9S_8$ before and after 100 h.

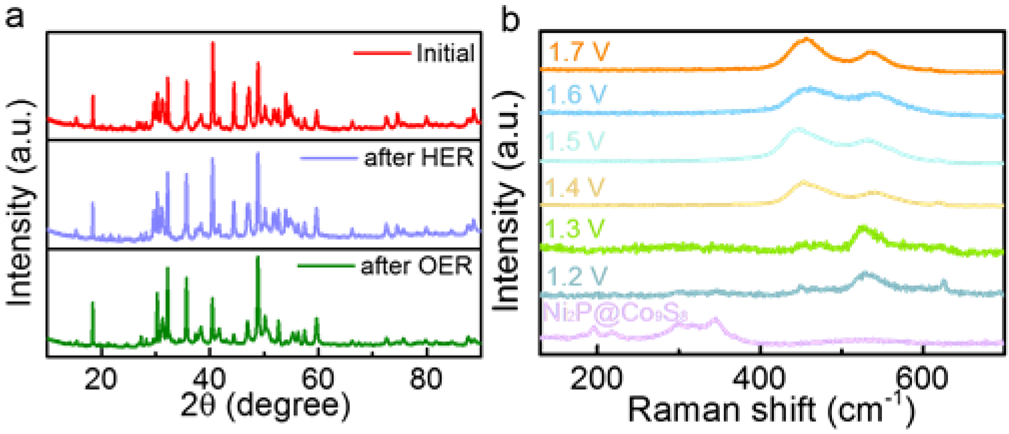

Figure S18 (a) XRD patterns of $Ni_2P@Co_9S_8$ before and after HER and OER reactions; (b) In situ Raman spectra of OER on $Ni_2P@Co_9S_8$ in 1 M KOH.

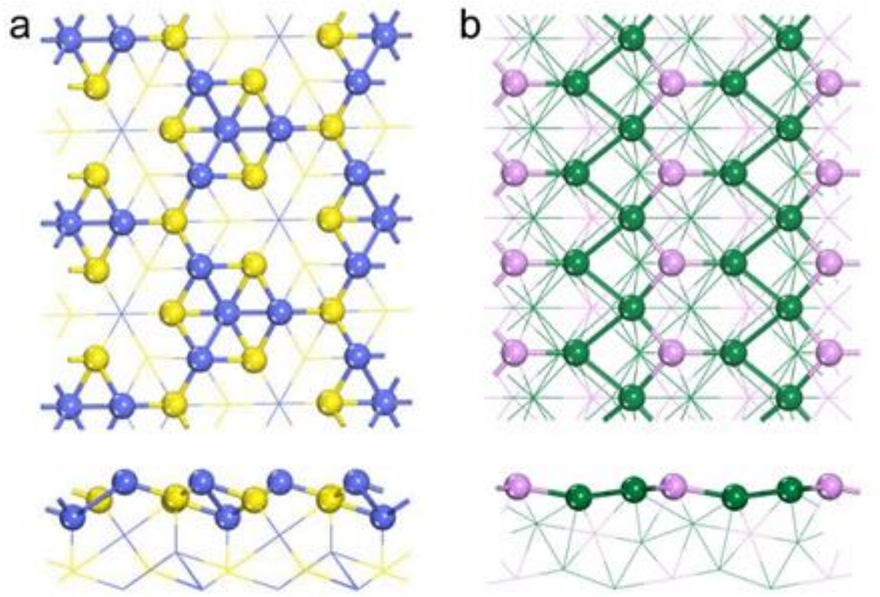

Figure S19 Optimized structures of (a) Co_9S_8(111) and (b) Ni_2P(100). The top layers are displayed in the ball-stick style while others are in the line style.

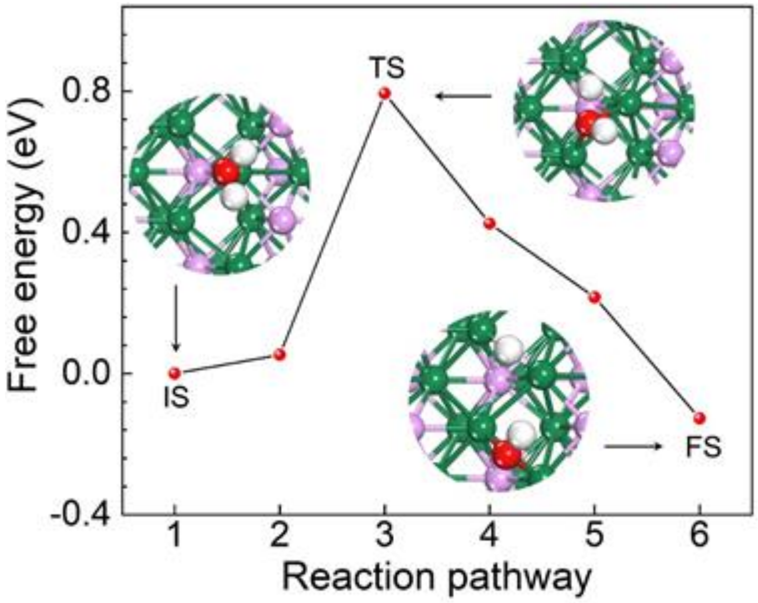

Figure S20 The energy barrier and reaction pathway of water dissociation on Ni_2P, including the corresponding configurations of IS, TS and FS.

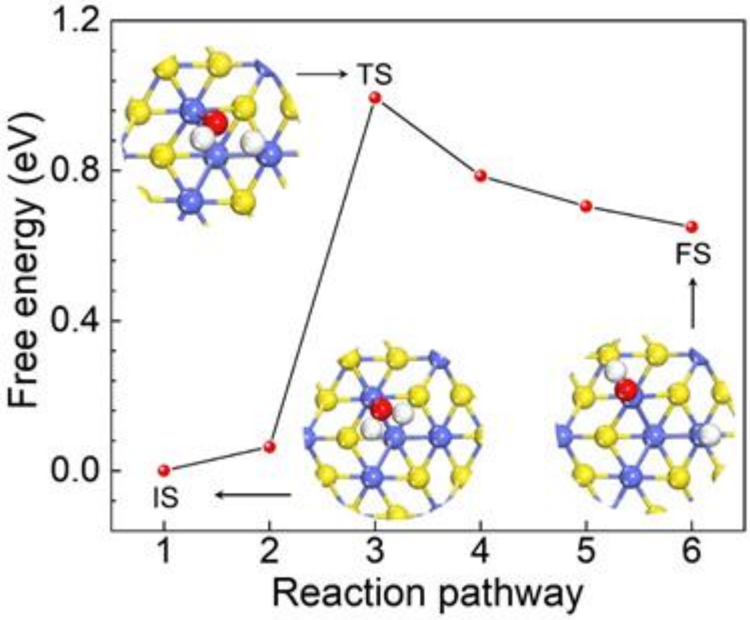

Figure S21 The energy barrier and reaction pathway of water dissociation on Co_9S_8, including the corresponding configurations of IS, TS and FS.

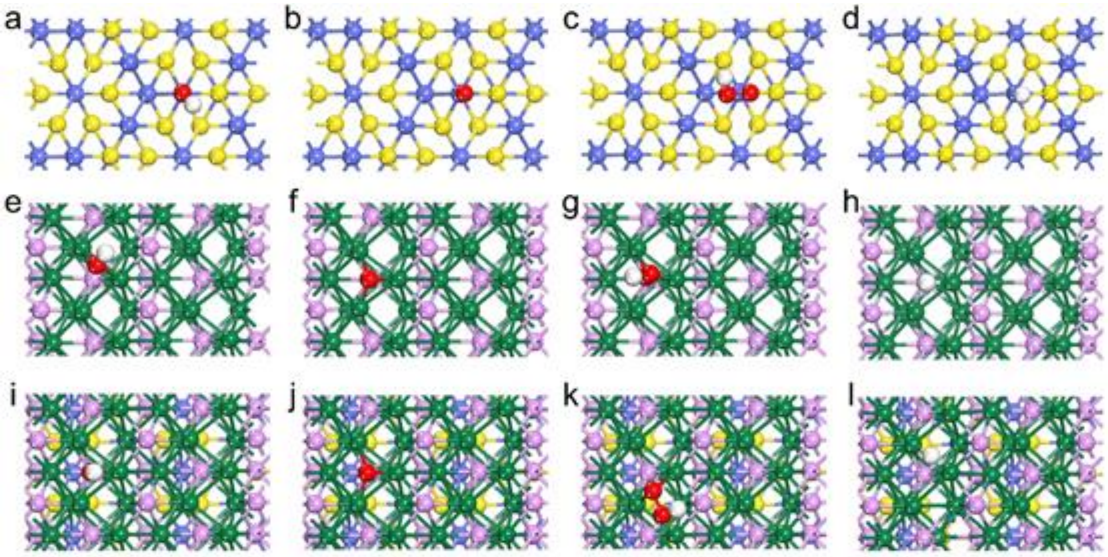

Figure S22 Top views of *OH, *O, *OOH and *H on (a-d) Co_9S_8, (e-h) Ni_2P and (i-l) $Ni_2P@Co_9S_8$.

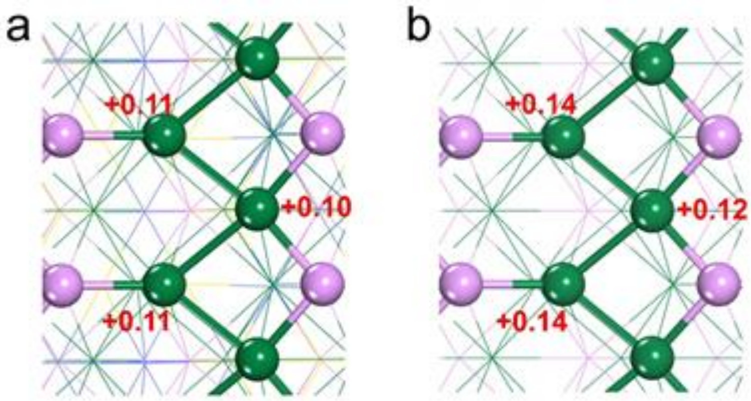

Figure S23 Bader charge analyses of (a) $Ni_2P@Co_9S_8$ and (b) Ni_2P including the Bader charge values of surface active Ni sites.

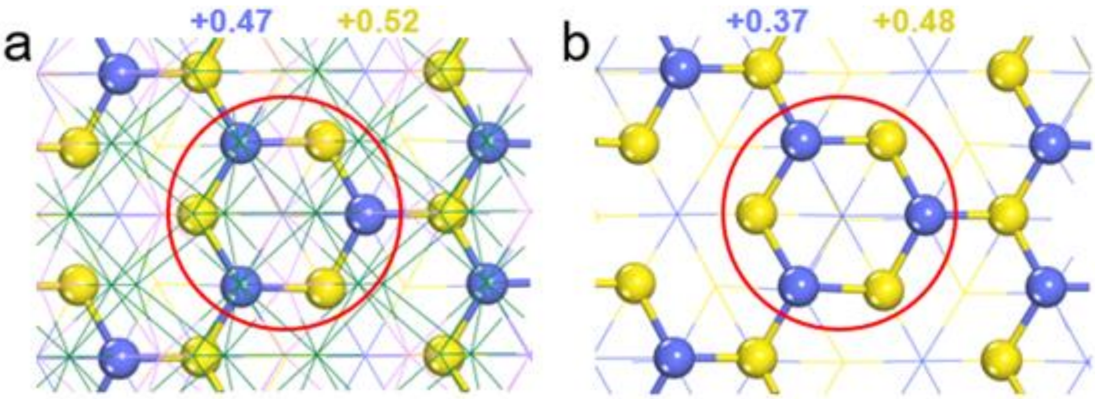

Figure S24 Bader charge analysis of topmost Co and S atoms in (a) $Ni_2P@Co_9S_8$ and (b) Co_9S_8. Target Co and S atoms are shown by red circles, and blue and yellow numbers are the average Bader charge values of three target Co and S atoms, respectively.

Table S1 Calculated double layer capacitance and corresponding RF values (HER).

Catalyst	C_{dl} (mF cm^{-2})	RF
$Ni_2P@Co_9S_8$	91	2275
Co_9S_8	54	1350
Ni_2P	55	1375

Table S2 R_{ct} values of $Ni_2P@Co_9S_8$, Co_9S_8 and Ni_2P for HER.

Catalyst	R_{ct} (Ω)
$Ni_2P@Co_9S_8$	2.79
Co_9S_8	3.52
Ni_2P	3.85

Table S3 Calculated double layer capacitance and corresponding RF values (OER).

Catalyst	C_{dl} (mF cm^{-2})	RF
$Ni_2P@Co_9S_8$	50	1250
Co_9S_8	28	700
Ni_2P	31	775

Table S4 R_{ct} values of $Ni_2P@Co_9S_8$, Co_9S_8 and Ni_2P for OER.

Catalyst	R_{ct} (Ω)
$Ni_2P@Co_9S_8$	2.51
Co_9S_8	3.06
Ni_2P	2.76

Table S5 Comparison of overpotentials at 100 mA cm^{-2} and Tafel slopes of different heterogeneous electrocatalysts recently reported for HER in 1.0 M KOH solution

Catalyst	Tafel (mV dec^{-1})	η (mV) @ 100 mA cm^{-2}	Reference
$Ni_2P@Co_9S_8$	70	188	This work
c-NiP_2/m-NiP_2	67	200	*Angew. Chem. Int. Ed.* ***2021****, 60, 259*
Ni_2P/MoS_2	60.2	260	*Adv. Funct. Mater.* ***2019****, 29, 1809151*
$ZnP@Ni_2P-NiSe_2$	82	214	*Adv. Funct. Mater.* ***2022****, 32, 2113224*
$Ni_5P_4-Ni_2P$ NS	79.1	200	*Angew. Chem. Int. Ed.* ***2015****, 54,* 8188-8192
$1T_{0.81}-MoS_2@Ni_2P$	79	292	*Nat. Commun.* ***2021****, 12, 5260*
NiCu@C	95	247	*J. Am. Chem. Soc.* ***2018****, 140, 610-617*
Ni_5Co_3Mo-OH	59	250	*ACS Energy Lett.* ***2019****, 4, 952-959*
Ni -ZIF/NiB	101	280	*Adv. Energy Mater.* ***2020****, 10, 1902714*
$Mo-Co_9S_8@C$	68	320	*Adv. Energy Mater.* ***2020****, 10, 1903137*
$PBA@Co(OH)_2$	100	260	*Adv. Energy Mater.* ***2019****, 9, 1802939*
NiFeSe	49	230	*Adv. Energy Mater.* ***2019****, 9, 1802983*
Ni-Mo-N/CFC	70	250	*Nat. Commun.* ***2019****,* 10, *5335*
$Co-Ni_3N$/CC	185	~256	*Adv. Mater.* ***2018****,* 30, *e1705516*
FeNiP-NPHC	102	182	*Adv. Funct. Mater.* ***2022****, 32, 2205767*
$Co_{0.42}Fe_{0.58}P@C$	66.6	181(10)	*Adv. Energy Mater.* ***2022****, 12, 2202394*

Table S6 Comparison of overpotentials at 100 mA cm^{-2} and Tafel slopes of different heterogeneous electrocatalysts recently reported for OER in 1.0 M KOH solution

Catalyst	Tafel (mV dec^{-1})	η (mV) @ 100 mA cm^{-2}	Reference
$Ni_2P@Co_9S_8$	57	253	This work
$MoS_2/Co_9S_8/Ni_3S_2/Ni$	58	420	*J. Am. Chem. Soc.* ***2019****, 141, 10417-10430*
Co_3O_4-NP/N-rGO	62	380	*Adv. Energy Mater.* ***2018*** *8, 1702222*
$CoFe_2O_4$@N-CNFs	80	349	*Adv. Sci.* ***2017****, 4, 1700226*
CF/VGSs/MoS	113	450	*Nat. Commun.* ***2021****, 12, 1380*
Co-MoS_2/BCCF-21	85	370	*Adv. Mater.* ***2018****, 30, 1801450*
Ni_2P-$NiSe_2$	71	326	*Adv. Funct. Mater.* ***2022****, 32, 2113224*
Fe-$Ni_2P@Cu_xS$	59	390	*ACS Energy Lett.* **2019***, 4, 952-959*
S-(Ni,Fe)OOH	48.9	281	*Energy Environ. Sci.* ***2020****, 13, 3439-3446*
$W_{0.5}Co_{0.4}Fe_{0.1}$/NF	310	42	*Angew. Chem. Int. Ed.* ***2017****, 56, 4502-4506*
2D/1D FeNi LDH/MOF	37.1	272	*Adv. Funct. Mater.* ***2021****, 31, 2103318*
Fe_2Ni-BPTC/CC	42	365	*Angew. Chem. Int. Ed.* ***2018****, 57, 9660*
(CrMnFeCoNi)S_x	66	295	*Adv. Energy Mater.* ***2021****, 11, 2002887*
CoMoNiS-NF-31	53	260	*J. Am. Chem. Soc.* ***2019****, 141, 10417-10430.*
MoOx/Ni_3S_2	72	310	*Adv. Funct. Mater.* ***2016****, 26, 4839.*
(Fe,Co)OOH/MI	73	290	*Adv. Mater.* ***2022****, 34, 2200270*
CoNiFeCu	43.5	292	*Adv. Mater.* ***2022****, 34, 2109108*
MoS_2/NiFe LDH‖MoNiFe	23	290	*Nat. Commun.* ***2022****, 13, 2191*

CoNiRu-NT	67	335	*Adv. Mater.* ***2022****, 34, 2107488*
FeCoNiMnRu/CNF	61.3	308	*Nat. Commun.* ***2022****, 13, 2662*
Mn-NG	55	337(10)	*Nat. Catal.* ***2018****, 1, 870-877*
Ni–NHGF	61	331(10)	*Nat. Catal.* ***2018****, 1, 63-72*

Table S7 Comparison of cell voltages at 10 and 100 mA cm^{-2} and stabilities of different bifunctional non−noble metal electrocatalysts recently reported for overall water splitting in 1.0 M KOH solution

Catalyst	Cell voltage at 10 mA cm^{-2} (V)	Cell voltage at 100 mA cm^{-2} (V)	Reference
$Ni_2P@Co_9S_8$	1.46	1.66	This work
FeCoNi/CC	1.55	2.00	*Adv. Energy Mater.* ***2019****, 9, 1901312*
Co_3S_4/MOF	1.55	1.90	*Adv. Mater.* ***2019****, 31, 1806672*
Ni/γ-Fe_2O_3	1.47	1.77	*Nat. Commun.* ***2019****, 10, 5599*
$CoFeZrO_x$	1.63	1.78	*Adv. Mater.* ***2019****, 31, 1901439*
Ni-ZIF/NiB	1.54	1.77	*Adv. Energy Mater.* ***2020****, 10, 1902714*
PBA@$Co(OH)_2$	1.65	1.99	*Adv. Energy Mater.* ***2019****, 9, 1802939*
Cr/FeNi-P	1.54	1.70	*Adv. Mater.* ***2019****, 31, 1900178*
CoMoNiS	1.54	2.09	*J. Am. Chem. Soc.* ***2019****, 141, 10417-10430*
Au/Ni_3S_2/NF	1.52	1.82*	*Appl. Catal. B: Environ.* ***2022****, 304, 120935*
MH-TMO	1.49	1.72	*Adv. Energy Mater.* ***2022,*** *12, 2200067*
BPIr-be	1.57	1.78	*Adv. Mater.* ***2021****, 33, 2104638*
O-CMMOFs-NF	1.47	1.85	*Appl. Catal. B: Environ.* ***2022****, 307, 121151*
Ni-MoO_2/NF-IH	1.50	1.72	*Adv. Funct. Mater.* ***2021****, 31, 2009580.*
CoFeO@BP	1.58	1.78	*Angew. Chem. Int. Ed.* ***2020****, 59, 21106.*
MoS_2/NiFe LDH‖MoNiFe	1.61	1.73	*Nat. Commun.* ***2022****, 13, 2191*

CoFeP@C	1.55	1.78	*Adv. Energy Mater.* ***2022****, 12, 2202394*
FeCoNi MOF	1.60	1.90	*J. Am. Chem. Soc.* ***2022****, 144, 3411*

Table S8 Comparison of the overpotentials at 200 mA cm^{-2} toward the HER in 1 M KOH of the $Ni_2P@Co_9S_8$ with other reported high-performance bifunctional catalysts.

Catalyst	η (mV) @ 200 mA cm^{-2}	Reference
$Ni_2P@Co_9S_8$	204	This work
$CoNC@Co_2N/CPs$	330	*Adv. Energy Mater.* ***2020****, 10, 2002214*
$Ni\text{-}N_3$	345	*Adv. Mater.* ***2020****, 33, 2003846*
$Cu1Ni_2\text{-}N$	255	*Adv. Energy Mater.* ***2019****, 9, 1900390*
$Ni_5Co_3Mo\text{-}OH$	290	*ACS Energy Lett.* ***2019****, 4, 952-959*
Cu_3N	335	*ACS Energy Lett.* ***2019****, 4, 747-754*
NiMoN/CFC	400	*Nat. Commun.* ***2019****, 10, 5335*
FeNiS/Ni	300	*Adv. Energy Mater.* ***2020****, 2001963*
NiFeP/graphene	290	*Adv. Mater.* ***2020****, 32, 1908201*
NiFeSe	325	*Adv. Energy Mater.* ***2019****, 9, 1802983*

Table S9 Comparison of the overpotentials at 200 mA cm^{-2} toward the OER in 1 M KOH of the $Ni_2P@Co_9S_8$ with other reported high-performance bifunctional catalysts.

Catalyst	η (mV) @ 200 mA cm^{-2}	Reference
$Ni_2P@Co_9S_8$	276	This work
NiCo LDH/NiCoS	378	*Nano Res.* ***2022****, 15, 4986-4995*
CoOOH	340	*J. Mater. Chem. A* ***2019****, 7, 7777-7783*
Co_2P-Co_3O_4	405	*Adv. Energy Mater.* ***2018****, 8, 1802445*
Ni/MoN/rNS)	533	*Adv. Sci.* ***2022****, 9, 2105869*
$NixCo_{3-x}S_4/Ni_3S_2$	318	*Nano Energy* ***2017****, 35, 161-170*
MoO_x/Ni_3S_2	312	*Adv. Funct. Mater.* ***2016****, 26, 4839-4847*
EG/$Co_{0.85}$Se/NiFe-LDH	290*	*Energy Environ. Sci.* ***2016****, 9, 478-483*
FeCoNiCuMn HEA/CNF	386	*Energy Environ. Sci.* ***2023****, 16, 619-628*
MX@MOF-Co_2P	407	*J. Mater. Sci. Technol.* ***2023****, 145, 74-82*
Ni/MoN/rNS	533	*Adv. Sci.* ***2022****, 9, 2105869*
Co/CoO/$Co(OH)_2$	360*	*Appl. Catal. B* ***2021****, 292, 120063*
Ir-1/$Ni_{1.6}Mn_{1.4}O_4$	350	*Adv. Sci.* ***2022****, 9, 2200529*

*The data were calculated according to the curves given in the literature

Table S10 Comparison of the overpotentials at 200 mA cm^{-2} toward the overall water splitting in 1 M KOH of the $Ni_2P@Co_9S_8$ with other reported high-performance bifunctional catalysts

Catalyst	Cell voltage at 200 mA cm^{-2} (V)	Reference
$Ni_2P@Co_9S_8$	1.76	This work
Co_3Se_4/CF	2.11	*Adv. Energy Mater.*, ***2017***, *7, 1602579*
CoP/NCNHP	1.97	*J. Am. Chem. Soc.* ***2018***, *140, 2610-2618*
NiCoP@NF-100	1.87	*J. Energy Chem.* ***2020***, *50, 395-401*
CoPO/NF	1.98	*Adv. Funct. Mater.* ***2018***, *28, 1706120*
NC@$CuCo_2N_x$/CF	1.93	*Adv. Funct. Mater.* ***2017***, *27, 1704169*
Ni-P/NF	2.17*	*J. Mater. Chem. A.* ***2016***, *4, 5639-5646*
Ni-P/CP	3.3*	*Adv. Funct. Mater.* ***2016***, *26, 4067-4077*
Cu_3N	1.8	*ACS Energy Lett.* ***2019***, *4, 747-754*
FeNiS/Ni	1.95	*Adv. Energy Mater.* ***2020***, *2001963.*
FeCoNi/CC	2.13	*Adv. Energy Mater.* ***2019***, *9, 1901312.*
NiFeP/graphene	1.90	*Adv. Mater.* ***2020***, *32, 1908201*

CoNC	1.91	*Energy Environ. Sci.* ***2020****, 13, 545-553*
FeCo/Co_2P@C	2.1	*Adv. Energy Mater.* ***2020****, 10, 1903854.*
CoNSC	1.78	*Adv. Energy Mater.* ***2020****, 10, 2002896*
NiCoP/NF	1.98	*Nano Lett.* ***2016****, 16, 7718-7725*

*The data were calculated according to the curves given in the literature
